# Employment status and mortality in the context of high and low regional unemployment levels in Belgium (2001–2011): A test of the social norm hypothesis across educational levels

**DOI:** 10.1371/journal.pone.0192526

**Published:** 2018-02-08

**Authors:** Deborah De Moortel, Paulien Hagedoorn, Christophe Vanroelen, Sylvie Gadeyne

**Affiliations:** 1 Department of Sociology, Interface Demography, Vrije Universiteit Brussel, Brussels, Belgium; 2 Research Foundation Flanders, Brussels, Belgium; 3 Centre for Health and Society, Institute of Medical Sociology, Medical Faculty, Heinrich-Heine-University Düsseldorf, Düsseldorf, Germany; 4 Health Inequalities Research Group (GREDS), Universitat Pompeu Fabra, Barcelona, Spain; Scientific Institute of Public Health (WIV-ISP), BELGIUM

## Abstract

Because of compositional effects (more highly educated unemployed) and differences in the vulnerability towards the health consequences of unemployment (i.e. disappointment paradox hypothesis and/or status inconsistency for highly educated unemployed), it is argued that indicators of educational attainment need to be included when investigating the social norm of unemployment. Data from the 2001 census linked to register data from 2001–2011 are used, selecting all Belgian employed and unemployed between 30 and 59-year-old at time of the census. Poisson multilevel modelling was used to account for clustering of respondents within sub-districts. For individuals with low education levels, the relative difference in mortality rate ratios between the unemployed and employed is smallest in those regions where aggregate unemployment levels are high. For highly educated, this social norm effect was not found. This study suggest that the social norm effect is stronger for workers with low education levels, while highly educated workers suffer from disappointment and status inconsistency.

## Introduction

The central objective of this study is to probe into the association between individual-level employment status and mortality in areas with high and low regional unemployment levels, taking the influence of a person’s educational attainment into account. Given the structurally high unemployment levels in many high-income countries and the increasing importance of education as a “labour market asset”, we believe that investigating the pathways between employment status, educational attainment and mortality is a timely endeavour.

It is well established that individual-level unemployment is associated with an increased risk of mortality [1–3]. However, the causal nature of the association between unemployment and mortality is not clear and a matter of debate [4–6]. Generally, two mechanisms are put forward to explain the relationship between unemployment and mortality: social causation and social selection. Social causation theory assumes that unemployment leads to bad health and death, because it entails a loss of income, poverty, social deprivation, adverse health-related behaviour and psychological stress [7]. In contrast, according to (direct) social selection theory, currently unemployed persons might have had health problems before they lost their job [2]. In this case, illness precedes unemployment [1]. In indirect health-related selection, people with higher risk factors for morbidity and mortality (e.g. adverse health behaviours) are also more inclined to lose their job [1,8].

A person’s educational attainment might influence the association between individual-level unemployment and mortality. An individual who simultaneously holds positions of unequal rank, such as having a high education but a low employment status (such as being unemployed) is referred to as status inconsistent [9]. Inconsistency of social status can be a structural source of stress in itself [10]. Previous research has shown a positive association between status inconsistency and all-cause mortality [11]. Therefore, highly educated unemployed might be particularly at risk of poor health/mortality. For such individuals with greater potential and expectations encountering economic adversity in adulthood (such as unemployment) is relatively unexpected, which potentially results in higher levels of stress and depression [12]. Consequently, the so-called *disappointment paradox hypothesis* might also lead us to expect a larger mortality gap between the unemployed and the employed among the highly educated compared to among individuals with low educational levels.

Contextual economic conditions, such as high or rising aggregate unemployment levels impact upon the association between individual employment status and mortality. Martikainen and Valkonen [13] showed that the association between individual-level unemployment and mortality tends to weaken during a period of rapidly increasing aggregate unemployment. A first explanation for this phenomenon refers to a compositional effect: unemployment becomes less health-selective when unemployment rates are high or rising. Under favourable economic circumstances, those who remain unemployed are a more selective group [2,14]. In this case, it can be expected that those with impaired health status (direct selection) and with higher risk factors for morbidity and mortality (indirect selection) are more likely than others to remain unemployed. In contrast, in the case of high or rising unemployment levels, individual-level unemployment may be experienced by a broader spectrum of the working population (including the more healthy) leading to a weakened association between health and individual-level unemployment [12].

An alternative explanation of the weakened association between individual-level unemployment and mortality in the context of high or rising unemployment levels refers to the reduced level of stigmatization when unemployment is widespread [2]. This so-called *social norm of unemployment hypothesis* assumes that if unemployment is widespread, one’s own unemployment represents a smaller deviation from the social norm [15]. This results in lower levels of stress and depression, partially moderating the damaging health effects of individual-level unemployment. Clark, Knabe and Rätzel [16] argued that the dividing line for the social-norm effect of aggregate unemployment is not employed vs. unemployed, but rather good vs. bad job prospects in general. The findings of Clark et al. [16] suggest that individuals with good job prospects will be more strongly negatively affected by high or rising levels of aggregate unemployment, while their poor-prospect counterparts will be less negatively, or even positively, affected [16].

Drawing on the findings of Clark et al. [16], the weakened association between individual-level unemployment and mortality when aggregate unemployment levels are high or rising, might be stronger for individuals with low education levels. The latter often have poor future job prospects and thus ‘benefit’ from the social norm [16]. Highly educated often have better future job prospects, and can thus be expected to experience negative effects from amongst others status inconsistency [9] and disappointment [12]. Moreover, we expect that the link between mortality and status inconsistency and disappointment will be more visible when unemployment levels are high or rising, because then the unemployed might notice that their working lives are influenced by macro-level forces, leading to amongst others feelings of despair [17].

The aim of this contribution is threefold. Firstly, this paper wishes to test the social norm of unemployment hypothesis by probing into the association between employment status and overall mortality when unemployment levels are regionally high and low. Secondly, it wants to test whether the social norm “effect” differs across educational groups; and thirdly, it investigates the disappointment paradox/status inconsistency hypotheses by focussing on relative mortality differences between unemployed and employed within different educational levels. The data used in this study consist of a unique linkage between the Belgian census 2001 and a mortality follow-up during 2001–2011. This is a unique dataset containing all deaths occurring in the entire Belgian population during the follow-up period. Aggregate unemployment levels are assessed by comparing different geographic areas in Belgium with high and low unemployment levels. No study so far has examined the triangular relationship between individual-level unemployment, educational attainment and mortality, while simultaneously taking variation in aggregate (regional) unemployment levels into account.

## Materials and methods

### Data design and study population

Analyses are based on exhaustive population-wide data, consisting of a linkage between the 2001 Belgian census and register information on survival status, emigration status and cause of death during the period 01/10/2001–31/12/2011. The study was approved by the Belgian Commission for the Protection of Privacy. The research population comprises all men and women aged 30 to 59 who were employed or unemployed and looking for a job in 2001. By focussing solely on those individuals who are out of work and actively seeking a job, we are following the definition of unemployment of the International Labour Organization (ILO). Individuals out of work and not looking for a job, students, pension recipients, etc. were excluded from the analyses. Limiting the analyses to the respondents aged 30 to 59 year excludes those individuals who are in lift-off or end-of-career periods and leads to a more homogenous study population. The sample size consists of 1,891,222 men (1,766,815 employed and 124,407 unemployed) and 1,457,325 women (1,279,902 employed and 177,423 unemployed) and 67,160 deaths in men and 25,654 deaths in women.

As there is great concern about selection effects in investigating the mortality impact of unemployment, only individuals in good and very good self-reported health at onset are included in the analyses. In this way, we partially avoid direct health selection into unemployment which enables us to concentrate on the mechanisms informed by the social norm theory. The sample size “in good health” consists of 1,547,319 men (1,476,252 employed and 71,067 unemployed) and 1,207,960 women (1,092,334 employed and 115,626 unemployed) and 42,851 deaths in men and 15,939 deaths in women.

#### Variables

**Educational attainment** was categorized according to the International Standard Classification of Education (ISCED): pre-primary and primary education (ISCED 0–1); lower secondary education (ISCED 2); upper secondary education (ISCED 3) and tertiary education (ISCED 4–6).

The geographic unit of analysis consists of **sub-districts**. Sub-districts are based upon the administrative district level (“arrondissement”) (N = 43) and the presence of an urban agglomeration. This administrative district level corresponds to the Eurostat Nomenclature of Territorial Units for Statistics (NUTS) level 3. Urban agglomerations are identified using the classification of Belgian metropolitan areas [18]. If an administrative district contains an urban agglomeration, that district is subdivided into an urban and a non-urban sub-district. Large metropolitan areas (such as Antwerp and Liege) are split into two urban sub-districts, one containing the inner city and one containing the surrounding agglomeration. The non-urban districts “Diksmuide and Veurne”, “Bastogne and Marche-en-Famenne”, “Arlon and Virton” are grouped together as they have a relatively small number of inhabitants. This ensures that each spatial unit contains at least 50,000 inhabitants. A total of 68 sub-districts are demarcated, of which 28 are classified as urban and 40 as non-urban.

The aggregate **unemployment rate** is calculated by dividing the number of unemployed individuals (both men and women aged 18–64) by the total labour force based on the 2001 census at the sub-district level. The unemployment rate is expressed in quartiles; each quartile includes an approximately equal number of sub-districts. The highest quartile consists of the districts with the highest unemployment rate and vice versa. The categorical variable ‘unemployment rate’ is included as a variable at sub-district level.

#### Control variables

All models additionally controlled for age, origin, living arrangements and a proxy for material wealth (housing conditions). Attained age was included as a continuous variable in the multivariable analyses and as a categorical variable (5-year age groups) in the descriptive analysis. Nationality of origin distinguished respondents with a Belgian origin, Western origin (i.e. all European countries and the highly industrialised/democratic countries US, Canada, Japan, Australia and New Zealand) and non-Western origin (i.e. immigrants from all other countries). Ethnic differences in mortality are established by many studies [19]. According to the healthy migrant hypothesis immigrants are more healthy than natives because it are the healthiest individuals that are selected into migration [20]. Living arrangement is based upon the LIPRO-classification, which holds information on the relationship status (single, in a married or cohabiting relationship) and on the presence of children in the household [21]. This results in the following categories: (i) single no children; (ii) single with children; (iii) couple no children; (iv) couple with children; and (v) other (i.e. cohabiting with parents, cohabiting with non-related adults who are not the partner, etc.). Living arrangements are an important predictor of mortality [22]. Being married is a protective factor, while being single is a risk factor for increased mortality [23]. Parents have lower mortality than childless individuals [24]. Housing conditions is included as a proxy for material wealth [25]. This indicator consists of a combination of ownership (tenant or owner) and comfort of the house (low-, mid- and high comfort dwellings), resulting in six categories. Comfort of the house is based on the number of large repairs needed, living space, number of bedrooms and amenities. In general, renters have higher mortality than owner-occupiers [25].

In total 35,906 men (2.3%) and 19,536 women (1.6%) have a missing value on education. Mortality rates for individuals with missing data on educational attainment are higher than that of the reference group (comparable to those of the pre-primary and primary education group) (results not show). In total 83 men (<0.1%) and 28 women (<0.1%) have a missing value on origin, while 81,933 men (5.3%) and 64,637 women (5.3%) have a missing value on housing quality. Including persons with missing values did not show different research results. Therefore, we excluded persons with missing values from the analysis. The final study population consists of 1,435,810 men (60,063 unemployed and 1,375,747 employed) and 1,127,323 women (103,383 unemployed and 1,023,940 employed) and 38,645 deaths in men and 14,531 deaths in women.

### Statistical analyses

First, mortality rate ratios (MRRs) are estimated for each variable in a bivariate model using random intercept Poisson multilevel regression models with individuals at level 1 and sub-districts at level 2. The log of the person-years is used as offset to account for differing exposure time.

Subsequently, three Poisson multilevel regression models are estimated. A first model includes all control variables and an interaction term between employment status and the aggregate unemployment level (using cross-classification). To investigate the social norm of unemployment hypothesis, we calculate the relative difference between the MRRs of the unemployed compared to the MRRs of the employed living in the same context of aggregate unemployment: $\frac{MRR\;Unemployed\;in\;Qx}{MRR\;Employed\;in\;Qx}$.

A second model includes all control variables and an interaction term between employment status and educational attainment (using cross-classification). To test the disappointment paradox/status inconsistency hypotheses, we calculate the relative difference between the MRRs of the unemployed compared to the MRRs of the employed with the same education level: $\frac{MRR\;Unemployed\;with\;educational\;level\;x\;}{MRR\;Employed\;with\;educational\;level\;x}.$

Then, three-way cross-level interaction terms (using cross-classification) are calculated between employment status, educational attainment and aggregate unemployment quartiles. To test whether the “social norm effect” only protects employed and unemployed with low education levels (individuals with poor prospects), we calculate the relative difference between the MRRs of the unemployed compared to the MRRs of the employed with the same education level and living in the same context of aggregate unemployment: $\frac{MRR\;Unemployed\;with\;educational\;level\;x\;in\;Qy}{MRR\;Employed\;with\;educational\;level\;x\;in\;Qy}$.

To further focus on the social norm effect, Poisson regression models were also estimated for each educational group separately. For this, the Poisson multilevel models (including all variables) are expanded by including an interaction term between employment status and unemployment-level quartiles. Based on the interaction term the relative difference in MRRs of the unemployed versus employed living in the same context of aggregate unemployment are calculated. Similarly, to further focus on the disappointment paradox/status inconsistency hypotheses, models were stratified by aggregate level of unemployment. Poisson regression models (no multilevel as we have too few level 2 units in each stratification) (including all variables) are expanded by including an interaction term between employment status and education. Based on the interaction term the relative difference in MRRs of the unemployed versus employed with the same education level are calculated. Essentially the stratified and pooled models test the same associations, but only the pooled analyses allow to formally test the significance of the third order effects. The advantage of running these (stratified) separate models is that the interactions are easier to comprehend.

To test the significance of the interaction effects, we calculated p-values of the likelihood-ratio (LR) test comparing models with and without interaction. To control for age, attained age is included in the models. Attained age accounts for the time-varying effect of age. Each individual’s total follow-up time is split into 5-year age groups by performing Lexis expansions to compute age-at-risk [26]. All models are estimated for men and women separately and were estimated using Stata^™^ version 14.2.

## Results

### Descriptive analyses

Descriptive statistics are presented in S1 and S2 Tables in the appendix. S1 Table presents the number of cases and the number of deaths for each of the variables included in the models. S2 Table shows the number of cases by included variables for employed and unemployed men.

Table 1 presents the results of the bivariate multilevel analyses. Unemployed men and women clearly have a higher mortality rate compared with employed men and women (MRR = 2.04; CI: 1.97–2.12, for men and 1.55; CI: 1.47–1.62, for women). Furthermore, the socio-economic variables show mortality gradients that confirm international research results. The highest mortality is indeed observed for individuals with lower levels of education, for people in more deprived housing conditions, for Belgians, for people in single-persons households and for those living in sub-districts belonging to the third and fourth unemployment quartiles.

**Table 1:** Bivariate analyses (MRRs), multilevel models, 4,203,945 person years for Men and 3,360,558 person years for Women.

	Men			Women		
	MRR	[95%	CI]	MRR	[95%	CI]
**Individual characteristics**						
*Employment status*						
Employed (ref.)	1			1		
Unemployed (looking for job)	2.04	1.97	2.12	1.55	1.47	1.62
*Age*	1.09	1.09	1.09	1.08	1.08	1.09
*Educational level*						
(Pre-)primary education	2.38	2.30	2.46	2.17	2.05	2.29
Lower secondary	1.73	1.68	1.78	1.66	1.59	1.74
Upper and post-secondary	1.28	1.24	1.31	1.27	1.22	1.33
Tertiary (ref.)	1			1		
*Origin*						
Belgian (ref.)	1			1		
Western	0.74	0.71	0.77	0.75	0.70	0.79
Not Western	0.50	0.46	0.53	0.62	0.55	0.70
*Living arrangements*						
Single no child(ren)	1.95	1.89	2.00	2.11	2.00	2.21
single with child(ren)	1.90	1.78	2.02	1.52	1.44	1.60
other	1.31	1.26	1.36	1.87	1.79	1.95
couple no child(ren)	1.78	1.73	1.82	1.45	1.35	1.57
couple with child(ren) (ref.)	1			1		
*Housing quality*						
Owner high	1			1		
Owner Mid	1.15	1.11	1.18	1.15	1.10	1.21
Owner Low	1.43	1.39	1.47	1.28	1.22	1.34
Tenant High	1.32	1.27	1.38	1.30	1.22	1.39
Tenant Mid	1.52	1.46	1.58	1.50	1.41	1.60
Tenant Low	1.89	1.82	1.95	1.61	1.53	1.71
**Sub-district characteristic**						
*Unemployment rate*						
Q1 (lowest) (ref.)	1			1		
Q2	0.95	0.92	0.97	0.99	0.94	1.03
Q3	1.19	1.16	1.23	1.20	1.14	1.26
Q4 (highest)	1.32	1.28	1.36	1.29	1.23	1.35

### Multivariable analyses

The results of the multivariable Poisson multilevel models for men and women are presented in Table 2 (interaction terms). The interaction effect between employment status and aggregated unemployment level shows that the mortality excess of the unemployed is highest for unemployed men in Q4 (MRR = 1.62; CI: 1.53–1.72) and for unemployed women in Q3 (MRR = 1.45; CI: 1.31–1.60), compared to employed in Q4 (see Model 1). When comparing the relative mortality differences between the unemployed and employed across levels of aggregate unemployment, smaller differences are observed in contexts with high unemployment levels than in contexts with low unemployment levels for women. For instance, the relative difference between employed and unemployed women in Q4 was smaller (MRR = 1.40; CI: 1.29–1.52) than in Q1 (MRR = 1.63; CI: 1.45–1.82). For men, this “social norm effect” was also found. The relative difference between employed and unemployed men in Q4 was smaller (MRR = 1.62; CI: 1.53–1.72) than in Q1 (MRR = 1.87; CI: 1.69–2.06). The p-values of the LR-tests are significant for women (p<0.05) and border significant for men (p = 0.06).

**Table 2:** Mortality rate ratios (MRR) and their 95% confidence intervals (CI) for all-cause mortality, men and women in good health aged 30–59 years, Belgium, 2001–2011*.

	Men			Relative difference^a^			Women			Relative difference^a^		
	MRR	CI		MRR	CI		MRR	CI		MRR	CI	
***Model 1 interaction model employment status and employment-level quartiles***												
Unemployed x unemployment rate Q1	1.36	1.19	1.55	1.87	1.69	2.06	1.36	1.21	1.54	1.63	1.45	1.82
x unemployment rate Q2	1.21	1.07	1.38	1.66	1.51	1.83	1.13	1.00	1.26	1.32	1.18	1.47
x unemployment rate Q3	1.61	1.44	1.80	1.76	1.64	1.89	1.45	1.31	1.60	1.50	1.37	1.65
x unemployment rate Q4	1.62	1.53	1.72	1.62	1.53	1.72	1.40	1.29	1.52	1.40	1.29	1.52
Employed x unemployment rate Q1	0.73	0.66	0.80				0.84	0.79	0.89			
x unemployment rate Q2	0.73	0.67	0.80				0.85	0.80	0.91			
x unemployment rate Q3	0.91	0.84	1.00				0.96	0.90	1.03			
x unemployment rate Q4	1						1					
p-values of LR-test comparing model with and without interaction						0.064						0.046
***Model 2 interaction model employment status and education***												
Unemployed x (pre-)primary education	2.52	2.34	2.72	1.55	1.43	1.67	1.79	1.60	1.99	1.32	1.17	1.48
x Low secondary education	2.36	2.20	2.52	1.59	1.49	1.70	1.79	1.65	1.94	1.49	1.37	1.61
x High Secondary education	2.43	2.26	2.60	1.84	1.71	1.97	1.70	1.56	1.86	1.44	1.32	1.56
x Tertiary education	1.97	1.80	2.15	1.97	1.80	2.15	1.63	1.44	1.83	1.63	1.44	1.83
Employed x (pre-)primary education	1.63	1.57	1.69				1.36	1.27	1.45			
x Low secondary education	1.48	1.44	1.53				1.20	1.14	1.26			
x High secondary education	1.32	1.28	1.36				1.19	1.14	1.24			
x Tertiary education (ref.)	1						1					
p-values of LR-test comparing model with and without interaction						0.000						0.092
***Model 3 interaction model employment status*. *education and unemployment level***												
Unemployed (pre-)primary education x Q1	2.04	1.64	2.54	1.71	1.39	2.09	1.61	1.24	2.08	1.52	1.16	1.99
xQ2	1.63	1.30	2.05	1.39	1.12	1.72	1.35	1.05	1.73	1.18	0.91	1.53
x Q3	2.50	2.11	2.95	1.73	1.49	2.01	1.85	1.49	2.30	1.40	1.11	1.77
x Q4	2.34	2.10	2.61	1.47	1.31	1.65	1.67	1.40	1.99	1.19	0.98	1.45
Unemployed low secondary education x Q1	1.82	1.49	2.23	1.71	1.42	2.05	1.66	1.37	2.01	1.68	1.38	2.04
xQ2	1.69	1.39	2.05	1.59	1.34	1.90	1.21	1.00	1.47	1.19	0.98	1.45
x Q3	2.38	2.05	2.77	1.76	1.56	1.99	1.87	1.60	2.18	1.58	1.36	1.84
x Q4	2.13	1.92	2.35	1.45	1.32	1.61	1.70	1.49	1.94	1.48	1.29	1.70
Unemployed high secondary education x Q1	1.79	1.46	2.21	1.91	1.58	2.31	1.64	1.35	2.01	1.65	1.35	2.00
xQ2	1.63	1.33	2.00	1.70	1.42	2.04	1.33	1.09	1.62	1.34	1.11	1.62
x Q3	2.07	1.76	2.43	1.73	1.50	1.98	1.57	1.32	1.86	1.39	1.18	1.65
x Q4	2.48	2.23	2.76	1.91	1.72	2.13	1.64	1.42	1.90	1.40	1.21	1.63
Unemployed tertiary education x Q1	1.71	1.33	2.20	2.39	1.89	3.03	1.39	1.00	1.93	1.67	1.20	2.33
xQ2	1.51	1.20	1.88	2.10	1.70	2.58	1.57	1.21	2.04	1.86	1.44	2.41
x Q3	1.63	1.34	1.99	1.83	1.53	2.19	1.44	1.14	1.81	1.56	1.24	1.95
x Q4	1.87	1.63	2.14	1.87	1.63	2.14	1.53	1.26	1.86	1.53	1.26	1.86
Employed (pre-)primary education x Q1	1.20	1.07	1.33				1.06	0.93	1.20			
xQ2	1.17	1.05	1.31				1.14	1.01	1.30			
x Q3	1.44	1.29	1.61				1.32	1.16	1.51			
x Q4	1.59	1.49	1.70				1.41	1.24	1.59			
Employed lower secondary education x Q1	1.06	0.96	1.18				0.99	0.89	1.09			
xQ2	1.06	0.95	1.17				1.02	0.92	1.13			
x Q3	1.35	1.22	1.49				1.18	1.07	1.30			
x Q4	1.46	1.38	1.55				1.15	1.05	1.26			
Employed higher secondary education x Q1	0.94	0.85	1.04				1.00	0.91	1.10			
xQ2	0.96	0.87	1.06				0.99	0.90	1.09			
x Q3	1.20	1.08	1.33				1.12	1.02	1.24			
x Q4	1.30	1.22	1.37				1.17	1.07	1.28			
Employed tertiary education x Q1	0.71	0.64	0.79				0.83	0.75	0.91			
xQ2	0.72	0.65	0.80				0.84	0.77	0.93			
x Q3	0.89	0.80	0.99				0.92	0.84	1.01			
x Q4 (ref.)	1						1					
p-values of LR-test comparing model with and without interaction						0.244						0.656

*All models are controlled for age, living arrangements, housing conditions and nationality of origin.

^a^ Model 1: employed in unemployment rate Q_x_ (ref.); Model 2: employed with educational level_x_ (ref.); Model 3: employed in unemployment rate Q_x_ with educational level_x_ (ref.).

Model 2 shows that there is a protective effect of education against the detrimental health effect of unemployment among men but not among women (Table 2). Unemployed men without tertiary education have a higher MRR than unemployed men with tertiary education. For women, although the MRR of unemployed with tertiary education is lower than that of the unemployed without tertiary education, this difference is not significant. The mortality excess of the unemployed compared to the employed with the same educational level is smaller for individuals with lower levels of education than for highly educated. For instance, the relative difference between employed and unemployed men with pre-primary and primary education is smaller (MRR = 1.55; CI: 1.43–1.67) than the relative difference between employed and unemployed men with tertiary education (MRR = 1.97; CI: 1.80–2.15). For women, this disappointment paradox and/or status inconsistency effect is also found, but the p-value of the LR-test indicates that the interaction between educational attainment and employment status is not significant.

Finally, the three-way interaction effects (employment status—education—unemployment level quartiles) provides more insight into the social norm hypothesis across educational levels. The highest mortality rates are found among unemployed men with pre-primary and primary education in Q3 (MRR = 2.50; CI: 2.11–2.95) and in unemployed women with low secondary education in Q3 (MRR 1.87; CI: 1.60–2.18) compared to the employed with tertiary education in Q4. Moreover, the relative differences in mortality between unemployed and employed do not significantly decrease when the unemployment levels increase across all educational groups. For instance, for men with high secondary education, the relative difference between employed and unemployed men in Q4 is not significantly smaller (MRR = 1.91; CI: 1.58–2.31) than in Q1 (MRR = 1.91; CI: 1.72–2.13).

### Social norm effect across educational groups

**Fig 1:**
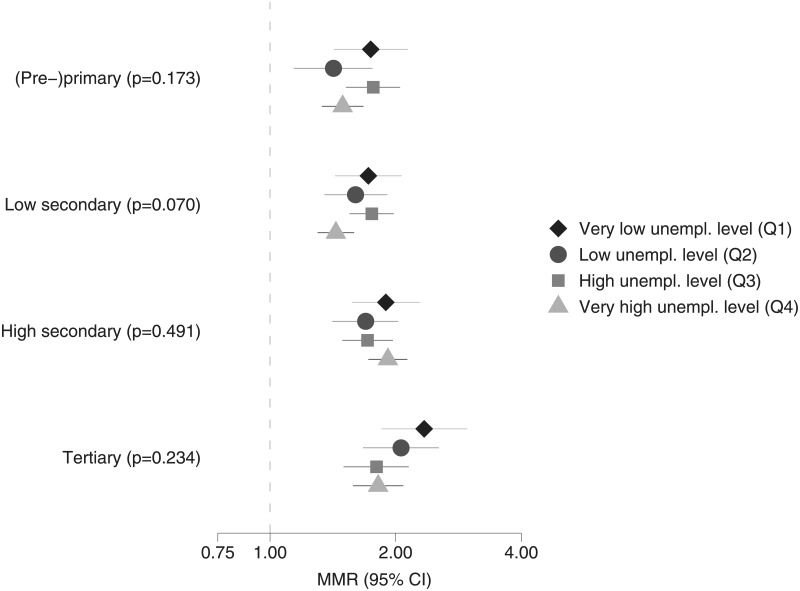
Relative differences in all-cause mortality, employed (reference group) versus unemployed living in the same area, for each educational level (including p-values for LR-tests comparing models with and without interaction), men in good health aged 30–59 years, Belgium, 2001–2011.

**Fig 2:**
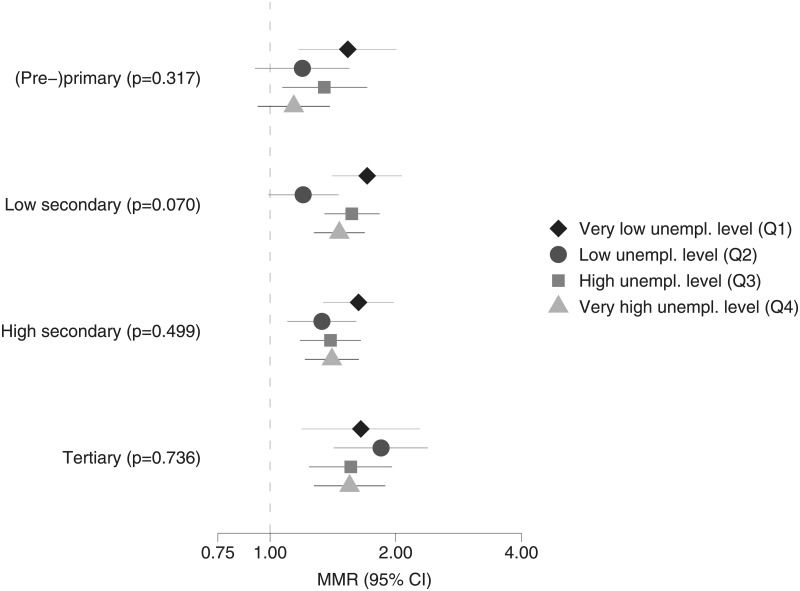
Relative differences in all-cause mortality, employed (reference group) versus unemployed living in the same area, for each educational level (including p-values for LR-tests comparing models with and without interaction), women in good health aged 30–59 years, Belgium, 2001–2011.

Next, Poisson multilevel models are estimated for each educational group separately. The relative differences in MRRs (based upon the interactions between employment status and employment-level quartiles) are shown per educational group for men in Fig 1 and for women in Fig 2. For both men and women, the relative differences in MRRs between employed and unemployed are generally larger in regions with very low unemployment levels, compared to regions with very high unemployment levels. However, most of the confidence intervals overlap and the p-values for the LR-test comparing the models with and without interactions are larger than 0.05. The p-values for the LR-test are lower for individuals with (pre-)primary and low secondary education, compared to individuals with high secondary and tertiary education. For individuals with low secondary education, the interactions are border significant (p = 0.07).

### Disappointment paradox/status inconsistency hypotheses across unemployment level quartiles

**Fig 3:**
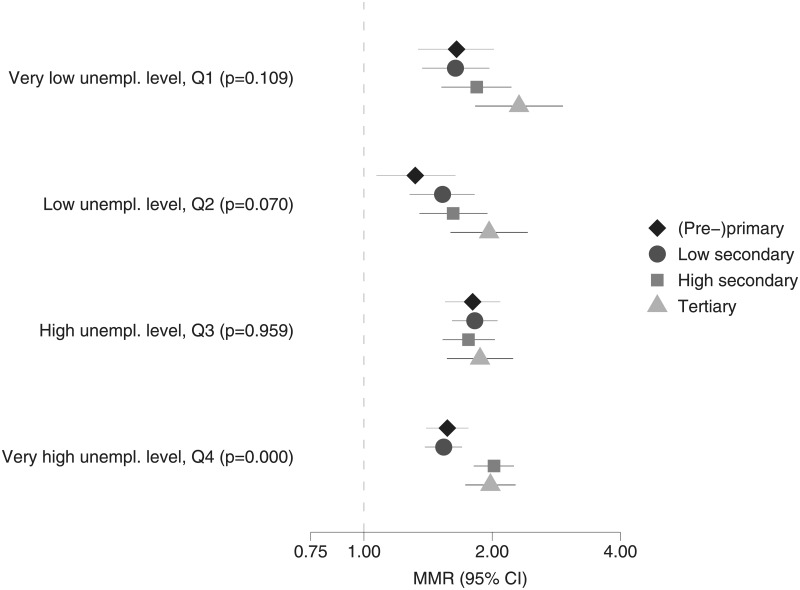
Relative differences in all-cause mortality, employed (reference group) versus unemployed with the same educational level, across regions with different unemployment rates (including p-values for LR-tests comparing models with and without interaction), men in good health aged 30–59 years, Belgium, 2001–2011.

**Fig 4:**
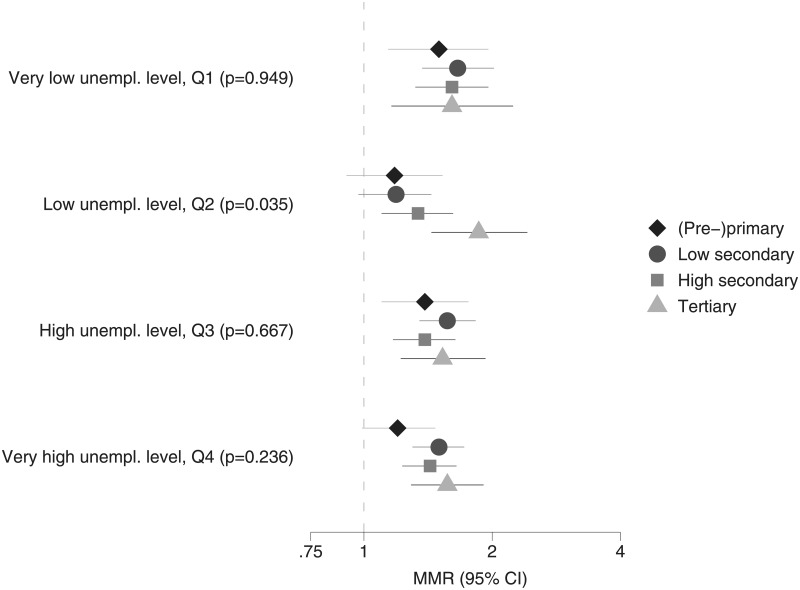
Relative differences in all-cause mortality, employed (reference group) versus unemployed with the same educational level, across regions with different unemployment rates (including p-values for LR-tests comparing models with and without interaction), women in good health aged 30–59 years, Belgium, 2001–2011.

The results of the analysis of the interaction effects between employment status and educational level within each quartile of aggregated unemployment are shown in Fig 3 for men and in Fig 4 for women. For men and women, in all contexts, the mortality excess of the unemployed is largest for individuals with tertiary education (in line with the disappointment paradox/status inconsistency hypothesises). However, differences are generally not significant as most confidence intervals overlap. Yet, for men in Q2, the interaction is border significant (p = 0.07). For men in Q4 and women in Q2, the interaction is significant (p≤0.05), showing that the disappointment paradox/status inconsistency hypotheses are influenced by aggregate unemployment levels.

## Discussion

Our analyses allow for four important conclusions: (1) the research results are in line with a social norm effect, showing a smaller mortality excess for unemployed men and women in regions with high aggregate unemployment levels; (2) this social norm effect is most obvious among the unemployed with low educational levels; (3) the results are in line with the disappointment paradox/status inconsistency hypotheses, showing the largest mortality excess for unemployed with tertiary education; and (4) the disappointment paradox/status inconsistency hypotheses are related to aggregate unemployment levels.

In line with other studies, we found that the unemployed have a higher risk of all-cause mortality than the employed [1,13]. Our results moreover suggest that the mortality excess of the unemployed is smaller when aggregate unemployment levels are high. This confirms the social norm of unemployment hypothesis and is consistent with earlier studies showing a weakened association between employment and mortality when aggregate unemployment levels are high [13]. In the case of high(er) aggregate unemployment levels, unemployed individuals probably encounter less stress as they might perceive a higher level of social support, shared empathy and understanding and a lower level of stigmatization [27]. However, reduced indirect (and direct) health selection might play a role too. When unemployment is experienced by a smaller spectrum of the workforce, individuals with increased risk factors for morbidity and mortality (such as unhealthy diets, smoking and/or drinking behaviour) might be more strongly represented among the unemployed [8]. When unemployment is widespread, the relative share of this group reduces as individuals with less risk factors for morbidity and mortality enter unemployment as well. In this study, we (partially) controlled for direct health selection, but were unable to control for indirect health selection. This might also explain the weakened association between employment status and mortality in the sub-districts with high aggregate unemployment levels.

Moreover, the social norm effect is most obvious for unemployed with low educational levels. The latter are probably to a higher degree protected by the social norm than highly educated unemployed. These results are in line with the findings of Clark et al. [16] who found that good-prospect unemployed are strongly negatively affected by regional unemployment levels.

The excess vulnerability of highly educated unemployed might be related to, amongst others, the disappointment paradox hypothesis [12] and the status inconsistency hypothesis [9]. Our results show that highly educated unemployed have a lower risk of all-cause mortality than unemployed with low educational levels. Yet, when comparing the relative mortality difference between unemployed and employed with the same educational level, we found that individuals with tertiary education show a larger (relative) difference in mortality than individuals with low educational levels. Thus, when individuals with tertiary education lose their job—assuming that selection effects are under control—they might experience the highest level of disappointment and/or status inconsistency. However, for women, mortality differences by employment status were not significantly different between educational groups.

Our findings show that the disappointment paradox hypothesis and the status inconsistency hypothesis are related to contextual conditions. Individuals with tertiary education are more vulnerable when aggregate unemployment levels are low. When few people are unemployed, it is difficult to retain a positive self-image as unemployment is less common and could be associated with personal failures [17]. However, our findings also suggest that men with tertiary education are more vulnerable when aggregate unemployment levels are very high. These men might notice that their working lives are influenced by macro-level forces that profoundly constrict their access to work, leading to amongst others feelings of despair [17].

### Strengths and limitations

This study highlights the importance of psychological responses (disappointment, stigmatization, etc.) to economic conditions. However, the reduced excess mortality for unemployed compared to employed with low educational levels could also be related to the adoption of healthier lifestyles during economic recessions, such as reducing smoking [28]. It has been shown that especially individuals with low socioeconomic positions have an increased risk of death because of unhealthy lifestyles and that economic recessions are associated with decreased mortality because of the increase in healthier lifestyles [28,29]. Unfortunately, we were unable to control for smoking behaviour or physical activity.

Nevertheless, the dataset used in this study is a unique dataset containing all Belgians and all deaths occurring during the period of follow-up. It consists of a unique linkage between the Belgian census 2001 and a mortality follow-up during 2001–2011. The data include extensive information on socioeconomic and socio-demographic characteristics, which made it possible to control models for housing conditions, living arrangements and origin and to include two- and three-way interaction terms. Future studies may tap into the investigated associations further by extending the follow-up period. Clarifying the role of career trajectories (including trajectories into and out of unemployment) for mortality by pooling different waves of census data might also be an interesting avenue for future research.

The use of multilevel analyses enabled us to study the variation in all-cause mortality, and the effects of exposure to aggregate unemployment levels. It takes the hierarchical structure of the data into account where individuals are nested within sub-districts, producing more reliable results [30]. These types of models are sensitive to the selection of the number of quadrature points. However, the use of a higher number of quadrature points (10 or 12), instead of the default seven quadrature points did not lead to different results. Alternative methods (involving fewer assumptions), such as population average models, can also be used to analyze hierarchical data [31].

Nevertheless, the use of sub-districts as geographical unit can be debated. Characteristics of small neighbourhoods and larger geographical units affect mortality too [32]. We used sub-districts as geographical unit to generate geographic areas containing enough cases (and deaths) to maintain stable estimates and to generate enough detail since the largest mortality effects are generally seen in small areas [32].

Usually when the influence of aggregate unemployment levels on the association between employment status and mortality is studied, evolutions across time are considered. In this study, the association is compared across areas with different aggregate unemployment levels which makes this a unique study. Using the total unemployed population in 2001 as an indicator of context-level unemployment can be debated as these levels might have changed during follow-up. However, including an average for the entire follow-up period at the level of sub-districts was not possible, as these data were not available. Regional variation in unemployment rates in Belgium is however relatively stable. Moreover, between 2001 and 2011, absolute unemployment rates in Belgium as a whole were quite stable as well: the lowest unemployment rate was 6 percent (2001) and the highest was 8 percent (2004 and 2009).

Another strength of this study is the focus on individuals who reported to be in good and very good health. Selection effects are of great concern in this research field. Therefore, we included only individuals in good health at baseline (2001) and excluded particular categories from the analyses (men who never worked, those who did not work because of social, family and health issues and the retired). However, we could not account for indirect health selection. That is, people with a latent risk profile or with personality traits that make them more prone for both higher mortality and unemployment [1,8]. We were not able to exclude these individuals from the dataset using the precautions mentioned above.

## Conclusion

In this study, we found evidence to support the social norm of unemployment, the disappointment paradox and the status inconsistency hypothesis. In addition, our results suggest that the social norm of unemployment is especially beneficial for unemployed with low education levels. More research is needed to further support the differential effect of the social norm across educational groups.

## Supporting information

S1 Table Number of cases and number of deaths by included variables, men and women in good health, aged 30 to 59, Belgium 2001. (DOCX)

S2 Table Number of cases by included variables, employed and unemployed men and women in good health, aged 30 to 59, Belgium 2001. (DOCX)
