# Supplementary material for: Employment status and mortality in the context of high and low regional unemployment levels in Belgium (2001–2011): A test of the social norm hypothesis across educational levels
Source: PLoS One. 2018 Feb 8;13(2):e0192526. doi: 10.1371/journal.pone.0192526 (PMC5805313; doi:10.1371/journal.pone.0192526)
Supplement: S1 Table — (DOCX) [file pone.0192526.s001.docx]

| **S1 Table. Number of cases and number of deaths by included variables, men and women in good health, aged 30 to 59, Belgium 2001** | | | | | | | | |
| --- | --- | --- | --- | --- | --- | --- | --- | --- |
|  | **Men** | | **Deaths in men** | | **Women** | | **Deaths in women** | |
|  | **N** | **%** | **N** | **%** | **N** | **%** | **N** | **%** |
| **Employment status** | |  |  |  |  |  |  |  |
| Employed | 1,375,747 | 95.82 | 35,473 | 2.58 | 1,023,940 | 90.83 | 12,552 | 1.23 |
| Unemployed | 60,063 | 4.18 | 3,172 | 5.28 | 103,383 | 9.17 | 1,979 | 1.91 |
| **Education** |  |  |  |  |  |  |  |  |
| (pre-)primary | 131,959 | 9.19 | 5,96 | 4.52 | 80,067 | 7.10 | 1,689 | 2.11 |
| Low secondary | 353,825 | 24.64 | 11,818 | 3.34 | 230,601 | 20.46 | 3,762 | 1.63 |
| High secondary | 475,095 | 33.09 | 11,676 | 2.46 | 378,883 | 33.61 | 4,714 | 1.24 |
| Tertiary | 474,931 | 33.08 | 9,191 | 1.94 | 437,772 | 38.83 | 4,366 | 1.00 |
| **Age at 2001** |  |  |  |  |  |  |  |  |
| 30-34 | 288,069 | 20.06 | 2,816 | 0.98 | 262,323 | 23.27 | 1,295 | 0.49 |
| 35-39 | 307,592 | 21.42 | 4,295 | 1.40 | 264,239 | 23.44 | 2,034 | 0.77 |
| 40-44 | 285,546 | 19.89 | 6,253 | 2.19 | 234,574 | 20.81 | 2,788 | 1.19 |
| 45-49 | 246,583 | 17.17 | 8,364 | 3.39 | 186,050 | 16.50 | 3,407 | 1.83 |
| 50-54 | 200,784 | 13.98 | 9,721 | 4.84 | 126,220 | 11.20 | 3,111 | 2.46 |
| 55-59 | 107,236 | 7.47 | 7,196 | 6.71 | 53,917 | 4.78 | 1,896 | 3.52 |
| **Origin** |  |  |  |  |  |  |  |  |
| Native | 1,231,393 | 85.76 | 34,529 | 2.80 | 989,114 | 87.74 | 13,129 | 1.33 |
| Western | 149,599 | 10.42 | 3,278 | 2.19 | 108,736 | 9.65 | 1,130 | 1.04 |
| Non-Western | 54,818 | 3.82 | 838 | 1.53 | 29,473 | 2.61 | 272 | 272 |
| **Living arrangement** | |  |  |  |  |  |  |  |
| Single no kids | 177,460 | 12.36 | 7,048 | 3.97 | 101,070 | 8.97 | 2,082 | 2.06 |
| Single with kids | 25350 | 1.77 | 1010 | 3.98 | 114,594 | 10.17 | 1,748 | 1.53 |
| Couple no kids | 242,485 | 16.89 | 8,832 | 3.64 | 175,924 | 15.61 | 3,196 | 1.82 |
| Couple with kids | 877,413 | 61.11 | 18,642 | 2.12 | 684,677 | 60.73 | 6,779 | 0.99 |
| other | 113,102 | 7.88 | 3,113 | 2.75 | 51,058 | 4.53 | 726 | 1.42 |
| **Housing conditions** | |  |  |  |  |  |  |  |
| Owner high | 575,736 | 40.10 | 12,471 | 2.17 | 464,300 | 41.19 | 5,032 | 1.08 |
| Owner mid | 253,695 | 17.67 | 6,306 | 2.49 | 202,947 | 18.00 | 2,533 | 1.25 |
| Owner Low | 289,337 | 20.15 | 9,083 | 3.14 | 210,619 | 18.68 | 2,935 | 1.39 |
| Tenant high | 91,124 | 6.35 | 2,482 | 2.72 | 78,313 | 6.95 | 1,102 | 1.41 |
| Tenant mid | 90,450 | 6.30 | 2,857 | 3.16 | 76,021 | 6.74 | 1,246 | 1.64 |
| Tenant Low | 135,468 | 9.43 | 5,446 | 4.02 | 95,123 | 8.44 | 1,683 | 1.77 |
| **Unemployment rate** | |  |  |  |  |  |  |  |
| Q1 | 386,117 | 26.89 | 9,526 | 2.47 | 293,678 | 26.05 | 3,429 | 1.17 |
| Q2 | 387,041 | 26.96 | 9,010 | 2.33 | 303,814 | 26.95 | 3,487 | 1.15 |
| Q3 | 342,332 | 23.84 | 9,944 | 2.90 | 268,777 | 23.84 | 3,734 | 1.39 |
| Q4 | 320,320 | 22.31 | 10,165 | 3.17 | 261,054 | 23.16 | 3,881 | 1.49 |
