# Supplementary material for: Employment status and mortality in the context of high and low regional unemployment levels in Belgium (2001–2011): A test of the social norm hypothesis across educational levels
Source: PLoS One. 2018 Feb 8;13(2):e0192526. doi: 10.1371/journal.pone.0192526 (PMC5805313; doi:10.1371/journal.pone.0192526)
Supplement: S2 Table — (DOCX) [file pone.0192526.s002.docx]

| **S2 Table. Number of cases by included variables, employed and unemployed men and women in good health, aged 30 to 59, Belgium 2001** | | | | | | | | |
| --- | --- | --- | --- | --- | --- | --- | --- | --- |
|  | **Unemployed Men** | | **Employed men** | | **Unemployed Women** | | **Employed women** | |
|  | **N** | **%** | **N** | **%** | **N** | **%** | **N** | **%** |
| **Education** |  |  |  |  |  |  |  |  |
| (pre-)primary | 12,182 | 20.28 | 119,777 | 8.71 | 14,896 | 14.41 | 65,171 | 6.36 |
| Low secondary | 18,243 | 30.37 | 335,582 | 24.39 | 33,725 | 32.62 | 196,876 | 19.23 |
| High secondary | 17,259 | 28.73 | 457,836 | 33.28 | 36,142 | 34.96 | 342,741 | 33.47 |
| Tertiary | 12,379 | 20.61 | 462,552 | 33.62 | 18,620 | 18.01 | 419,152 | 40.94 |
| **Age at 2001** |  |  |  |  |  |  |  |  |
| 30-34 | 14,996 | 24.97 | 273,073 | 19.85 | 27,646 | 26.74 | 234,677 | 22.92 |
| 35-39 | 12,914 | 21.50 | 294,678 | 21.42 | 24,534 | 23.73 | 239,705 | 23.41 |
| 40-44 | 10,771 | 17.93 | 274,775 | 19.97 | 19,351 | 18.72 | 215,223 | 21.02 |
| 45-49 | 9,215 | 15.34 | 237,368 | 17.25 | 15,507 | 15.00 | 170,543 | 16.66 |
| 50-54 | 7,345 | 12.23 | 193,439 | 14.06 | 10,571 | 10.23 | 115,649 | 11.29 |
| 55-59 | 4,822 | 8.03 | 102,414 | 7.44 | 5,774 | 5.59 | 48,143 | 4.70 |
| **Origin** |  |  |  |  |  |  |  |  |
| Native | 38,030 | 63.32 | 1,193,363 | 86.74 | 75,887 | 73.40 | 913,227 | 89.19 |
| Western | 10,372 | 17.27 | 139,227 | 10.12 | 17,617 | 17.04 | 91,119 | 8.90 |
| Non-Western | 11,661 | 19.41 | 43,157 | 3.14 | 9,879 | 9.56 | 19,594 | 1.91 |
| **Living arrangement** | |  |  |  |  |  |  |  |
| Single no kids | 19,041 | 31.70 | 158,419 | 11.52 | 9,610 | 9.30 | 91,460 | 8.93 |
| Single with kids | 1,969 | 3.28 | 23,381 | 1.70 | 21,105 | 20.41 | 93,489 | 9.13 |
| Couple no kids | 8,520 | 14.19 | 233,965 | 17.01 | 14,990 | 14.50 | 160,934 | 15.72 |
| Couple with kids | 22,739 | 37.86 | 854,674 | 62.12 | 53,069 | 51.33 | 631,608 | 61.68 |
| other | 7,794 | 12.98 | 105,308 | 7.65 | 4,609 | 4.46 | 46,449 | 4.54 |
| **Housing conditions** | |  |  |  |  |  |  |  |
| Owner high | 10,331 | 17.20 | 565,405 | 41.10 | 28,278 | 27.35 | 436,022 | 42.58 |
| Owner mid | 6,164 | 10.26 | 247,531 | 17.99 | 15,568 | 15.06 | 187,379 | 18.30 |
| Owner Low | 11,148 | 18.56 | 278,189 | 20.22 | 19,418 | 18.78 | 191,201 | 18.67 |
| Tenant high | 4,780 | 7.96 | 86,344 | 6.28 | 9,908 | 9.58 | 68,405 | 6.68 |
| Tenant mid | 7,863 | 13.09 | 82,587 | 6.00 | 11,288 | 10.92 | 64,733 | 6.32 |
| Tenant Low | 19,777 | 32.93 | 115,691 | 8.41 | 18,923 | 18.30 | 76,200 | 7.44 |
| **Unemployment rate** | |  |  |  |  |  |  |  |
| Q1 | 6,844 | 11.39 | 6,844 | 27.57 | 15,086 | 14.59 | 278,592 | 27.21 |
| Q2 | 9,164 | 15.26 | 377,877 | 27.47 | 21,157 | 20.46 | 282,657 | 27.60 |
| Q3 | 14,785 | 24.62 | 327,547 | 23.81 | 26,900 | 26.02 | 241,877 | 23.62 |
| Q4 | 29,270 | 48.73 | 291,050 | 21.16 | 40,240 | 38.92 | 220,814 | 21.57 |
